# pH-Dependent selective extraction of gold(iii) from synthetic solution and computer motherboard leachate using a hybrid nanocomposite†

**DOI:** 10.1039/d4ra04476b

**Published:** 2024-07-17

**Authors:** Rabeea D. Abdel-Rahim, Mahmoud Thabet, Ahmed R. Abdellah, Mohamed O. Saleh, Ahmed M. M. Fadl, Adham M. Nagiub, Hassanien Gomaa

**Affiliations:** a Department of Chemistry, Faculty of Science, Al-Azhar University Assiut 71524 Egypt h.gomaa@azhar.edu.eg

## Abstract

Recycling gold from electronic waste offers significant benefits for both environmental protection and resource sustainability. However, this process presents considerable challenges due to high costs, prolonged processing times, and interference from coexisting metals. In this study, we synthesized a hybrid mesoporous nanocomposite comprising platelets-like CoNi_2_S_4_ incorporated with g-C_3_N_4_ nanosheets (CoNi_2_S_4_@g-C_3_N_4_) for the selective recovery of gold (Au(iii)) ions from spent computer motherboards. Comprehensive characterization of the CoNi_2_S_4_@g-C_3_N_4_ nanocomposite was conducted, including its physicochemical properties, textural and structural characteristics, morphology, and elemental composition. The CoNi_2_S_4_@g-C_3_N_4_ extractor demonstrated an exceptional adsorption capacity of 200.6 mg g^−1^, with high selectivity at pH 2, rapid equilibrium time of 60 minutes, and satisfactory reusability for over ten cycles. Adsorption isotherm and kinetic studies revealed that the CoNi_2_S_4_@g-C_3_N_4_ nanocomposite adheres to the Langmuir adsorption model and the pseudo-second-order kinetic model for Au(iii) ion adsorption. Overall, this study introduces a viable adsorbent that shows considerable promise for industrial-scale Au(iii) extraction from e-waste.

## Abstract 2

Recycling gold from electronic waste offers significant benefits for both environmental protection and resource sustainability.

## Graphical abstract

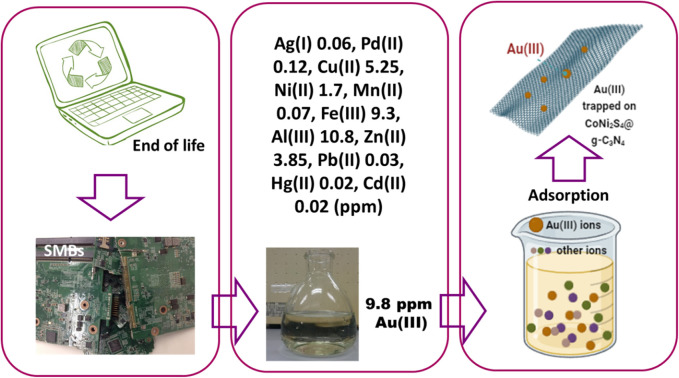

## 1. Introduction

Electronic waste (e-waste) represents a rapidly expanding solid waste stream, projected to reach 61.3 million tons annually by 2023.^1,2^ Unlike conventional solid wastes, e-waste contains a unique combination of hazardous and resource-rich materials, with only 17.4% currently being effectively recovered due to underdeveloped technologies.^3–5^ Spent motherboards (SMBs) from obsolete computers are a significant category of e-waste, containing valuable base metals (*e.g.*, copper, lead, tin) and precious metals (*e.g.*, gold, silver, palladium).^6^ Among these, gold (Au) is particularly valuable due to its extensive use in electronics, telecommunications, aerospace, and other high-tech industries.^7–10^ Efficient recovery of Au(iii) ions from SMBs is crucial for sustainable resource utilization and environmental protection.^11,12^ Therefore, efficient and sustainable technologies for Au(iii) recovery from SMBs must be designed.

Methods for Au(iii) recovery from e-waste involve pyrometallurgy and hydrometallurgy. A pyrometallurgical method often involves directly burning e-waste, which can produce by-pollutants and harmful materials. In contrast, the hydrometallurgical method provides a more environmentally friendly approach by utilizing digestive liquids to dissolve e-waste components before recovering the aimed species. The obtained solution undergoes subsequent extraction and purification processes.^13,14^ Various gold extraction technologies, such as ion exchange, precipitation, flotation, solvent extraction, and adsorption, have been developed.^15^ However, many of these approaches have noteworthy drawbacks, including high reagent requirements, the use of poisonous chemicals, and the production of toxic by-waste that necessitates discarding.^16,17^ Among these methods, adsorption stands out as the most reliable technique for gold extraction due to its low cost, simplicity, efficiency, and reusability.^18,19^ Recent developments have led to the creation of various adsorbents for trapping Au(iii) ions, including biomass materials,^20^ ion imprinting polymers,^21,22^ carbon-based materials,^23^ and metal–organic frameworks.^21^ However, many of these adsorbents suffer from limitations such as lower adsorption capacities, slower kinetics, and reduced selectivity in the presence of competing ions. In this manuscript, we aim to design a stable adsorbent with a high adsorption capacity and selectivity feature for Au(iii)-recovery from e-waste, even in the existence of other competing ions.

In recent years, graphitic carbon nitride (g-C_3_N_4_) has gained significant attention due to its unique physicochemical properties, including high chemical stability, higher electronic characteristics, plentiful functional groups, efficient visible light absorption for photocatalytic reactions, and acceptable adsorption capacity.^22–24^ In addition, g-C_3_N_4_ has plenty of π-conjugated electrons and nitrogen-containing active sites, enabling the formation of covalent bonds with cations such as Au(iii) ions. Despite these advantages, g-C_3_N_4_'s adsorption performance is often limited by its relatively low surface area and restricted pore structure. Various strategies have been employed to enhance g-C_3_N_4_'s adsorption properties, including texture modification, elemental manipulation, and incorporation with other materials.^25^ Introducing metal sulfides, particularly binary metal sulfides such as CoNi_2_S_4_, offers a promising approach to enhance the adsorption performance of g-C_3_N_4_. Metal sulfides exhibit improved surface morphology, electrical conductivity, thermal stability, and diverse redox properties, leading to efficient Au(iii) trapping.^26,27^ Binary metal sulfide compounds exhibit superior performance compared to single metal sulfides.^28,29^ However, to the best of our knowledge, the direct incorporation of CoNi_2_S_4_ into the g-C_3_N_4_ matrix to trap and extract Au(iii) ions from e-waste has not been previously reported.

This study introduces a selective recovery method for Au(iii) ions from e-waste-derived SMBs utilizing a mesoporous CoNi_2_S_4_@g-C_3_N_4_ nanocomposite. We comprehensively evaluated the Au(iii) adsorption properties of the CoNi_2_S_4_@g-C_3_N_4_ nanocomposite compared to the original g-C_3_N_4_. The report encompasses four main aspects: (i) synthesis and detailed characterization of g-C_3_N_4_ and CoNi_2_S_4_@g-C_3_N_4_ nanocomposites, (ii) investigation of the Au(iii) adsorption properties of g-C_3_N_4_ and CoNi_2_S_4_@g-C_3_N_4_ adsorbents, considering variables such as solution pH, contact time, sorbent dosage, Au(iii) concentration, and the presence of interfering ions typically found in e-waste leachates, (iii) assessment of the materials for Au(iii) recovery from actual SMB leachate, demonstrating their practical utility, and (iv) discussion on the Au(iii) adsorption mechanisms using isotherm and kinetic models to understand the adsorbents-Au(iii) interaction. Our findings indicate that the mesoporous CoNi_2_S_4_@g-C_3_N_4_ extractor shows considerable promise for selectively extracting gold from SMBs. This innovative approach not only enhances the efficiency of the recovery process but also contributes to the development of sustainable technologies for resource utilization and environmental protection.

## 2. Experimental work

### 2.1. g-C_3_N_4_ synthesis

**Fig. 1:**
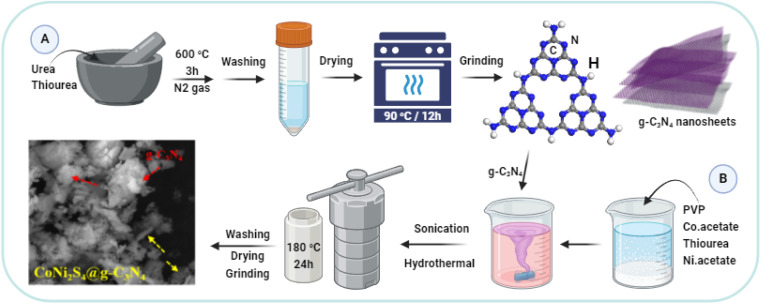
Schematic design for g-C_3_N_4_ (A) and CoNi_2_S_4_@g-C_3_N_4_ synthesis (B).

g-C_3_N_4_ was synthesized through a method involving the equal proportion mixing of urea and thiourea, followed by thorough grinding. A mixture of 10 g of urea and 10 g of thiourea was thoroughly ground and placed in a covered porcelain crucible. The crucible was then calcined at 600 °C for 3 hours in a muffle furnace under a nitrogen atmosphere. The resulting yellow g-C_3_N_4_ product was washed with deionized water and dried at 90 °C for 12 hours. The yield was approximately 35% (Fig. 1A).

### 2.2. Synthesis of CoNi_2_S_4_@g-C_3_N_4_

The CoNi_2_S_4_@g-C_3_N_4_ composite was fabricated through a multi-step process (Fig. 1B). Initially, polyvinylpyrrolidone (PVP, M wt 55 000 g mol^−1^) weighing 0.5 grams was dissolved in 40 mL of deionized water under continuous stirring. To this solution, 2.18 grams of Co(C_2_H_3_O_2_)_2_·4H_2_O was added, followed by a stirring period of 30 minutes. Subsequently, 1.52 grams of thiourea was incorporated into the mixture, which was then followed by the addition of 2.48 grams of Ni(C_2_H_3_O_2_)_2_·4H_2_O. The obtained solution was stirred for a further 30 minutes. Thereafter, 0.5 grams of pre-synthesized g-C_3_N_4_ was introduced to the solution and homogenized using ultrasonic agitation for a duration of 60 minutes. The homogenized mixture was then moved to a sealed autoclave for hydrothermal treatment at a temperature of 180 °C for 24 hours. The resulting black precipitate was isolated and thoroughly cleaned with deionized water three times to eliminate impurities. The final CoNi_2_S_4_@g-C_3_N_4_ product was obtained after drying at 75 °C overnight and was subsequently utilized for Au(iii) adsorption experiments. The yield of CoNi_2_S_4_@g-C_3_N_4_ was approximately 82% based on the total weight of g-C_3_N_4_ and metal precursors used.

### 2.3. Au(iii) adsorption methodology

A series of batch trials were performed to investigate the adsorption capacity of Au(iii) onto g-C_3_N_4_ and CoNi_2_S_4_@g-C_3_N_4_. These experiments involved stirring 25 mL of a 30 ppm Au(iii) solution with varying amounts of the adsorbent materials at room temperature, with the pH adjusted to a specific value. Small amounts of 0.1 M HCl or NaOH solutions were added as micro-drops to alter the pH of solutions. Following the stirring process, the samples were filtered, and the remaining Au(iii) concentrations were measured using Atomic Absorption Spectroscopy. To construct adsorption isotherms, 20 mg of the adsorbents were added to 25 mL of an Au(iii) solution of known concentration and stirred for 60 minutes at a pH of 2.0. Additionally, the influence of contact time on adsorption efficiency was assessed by introducing 20 mg of the adsorbent into 25 mL of a 30 ppm Au(iii) solution, maintaining the temperature and pH at room conditions and 2.0, respectively, and conducting periodic analyses. The adsorption performance of g-C_3_N_4_ and CoNi_2_S_4_@g-C_3_N_4_ and the Au(iii)-adsorbed amount (*q*_e_, mg g^−1^) were calculated by determining the difference in concentration before and after the adsorption process, using the following equations:^30,31^
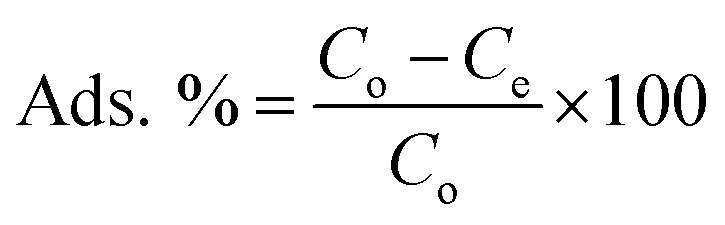

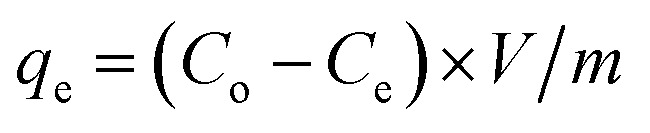


*C*_o_ and *C*_e_ (ppm) represent the original and final concentrations of Au(iii), respectively. The dose of the g-C_3_N_4_ and CoNi_2_S_4_@g-C_3_N_4_ adsorbents is denoted as *m* (g), and *V* (L) refers to the Au(iii) solution volume. The adsorbed Au(iii) ions could be released/eluted using an eluent agent, such as NaOH combined with thiourea, through a batch protocol. The elution proficiency was calculated using the next equation:^32,33^
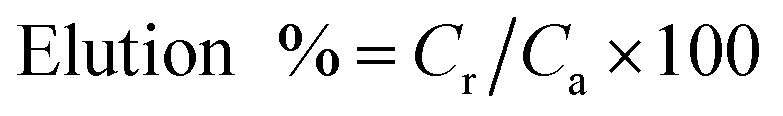


*C*_r_ and *C*_a_ (ppm) represent the concentrations of released and adsorbed Au(iii) ions, respectively. The recycled g-C_3_N_4_ and CoNi_2_S_4_@g-C_3_N_4_ adsorbents were reused for up to 10 adsorption–elution cycles using a batch design. The practical applicability of g-C_3_N_4_ and CoNi_2_S_4_@g-C_3_N_4_ adsorbents was tested by extracting Au(iii) ions from the leach liquor of e-waste. Each experiment was performed in triplicate and repeated thrice to guarantee reproducibility.

## 3. Outcomes and discussion

### 3.1. Adsorbents characterization

**Fig. 2:**
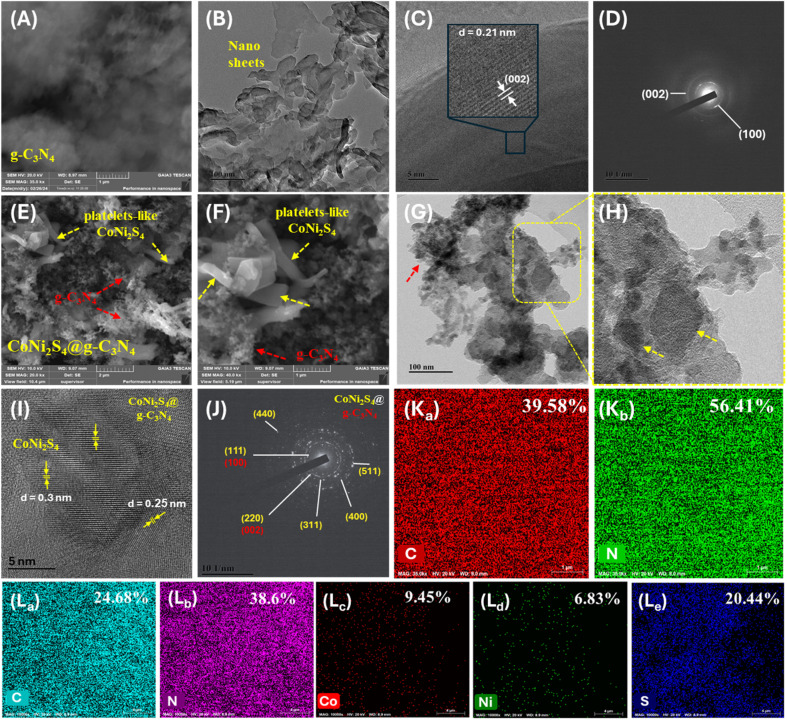
SEM (A), TEM (B), HRTEM (C), and SAED images (D) of the g-C_3_N_4_ sample; SEM (E and F), TEM (G and H), HRTEM (I), SAED images (J) of CoNi_2_S_4_@g-C_3_N_4_; elemental mapping of g-C_3_N_4_ (K_a–b_) and FeNi_2_S_4_@g-C_3_N_4_ (L_a–e_) samples.

To investigate the morphology and microstructure of the g-C_3_N_4_ sample, scanning electron microscopy (SEM) images (Fig. 2A) reveal a nanosheet-like morphology, with these sheets assembling into large blocks approximately 30 μm in size. Transmission electron microscopy (TEM) images (Fig. 2B) show g-C_3_N_4_ nanosheets with widths and lengths ranging from 50 to 100 nm and ultrathin thicknesses. Darker regions in the TEM images indicate the overlap of several few-layer g-C_3_N_4_ sheets, enhancing the diffusion and trapping performance of Au(iii) ions. High-resolution TEM (HRTEM) images (Fig. 2C) of g-C_3_N_4_ reveal uniformly distributed lattice fringe distances of 0.21 nm corresponding to the (002) plane. Selected area electron diffraction (SAED) patterns (Fig. 2D) show two concentric rings, confirming the crystal structure and corresponding to the (100) and (002) planes of g-C_3_N_4_. The morphology of the CoNi_2_S_4_@g-C_3_N_4_ sample was examined using SEM and TEM (Fig. 2E–H). SEM images (Fig. 2E and F) display platelets-like CoNi_2_S_4_ incorporated with g-C_3_N_4_ nanosheets. TEM images (Fig. 2G and H) illustrate the grafting of CoNi_2_S_4_ platelets among the sheet-like surfaces of g-C_3_N_4_. HRTEM analysis (Fig. 2I) indicates cubic CoNi_2_S_4_ planes and g-C_3_N_4_, with inter-planar spacings of 0.3 nm, 0.25 nm, and 0.28 nm corresponding to the (311), (400), and (440) crystal planes of CoNi_2_S_4_ nanoparticles, respectively. SAED patterns (Fig. 2J) further confirm the successful formation of the CoNi_2_S_4_@g-C_3_N_4_ nanocomposite by showing several diffraction rings that correspond to the lattice planes of both CoNi_2_S_4_ crystal and g-C_3_N_4_. Mapping of ingredients and energy-dispersive X-ray spectroscopy (EDS) profiles of g-C_3_N_4_ (Fig. 2K and S1†) demonstrate the homogenous distribution of C, N, and S elements throughout the surface. The atomic percentages of C, N, and S are 39.58%, 56.41%, and 4.01%, respectively, with the presence of sulfur attributed to the use of thiourea during synthesis. Elemental mapping images (Fig. 2L) of CoNi_2_S_4_@g-C_3_N_4_ show a homogeneous distribution of C, N, Co, Ni, and S elements, with atomic percentages of 24.68%, 38.6%, 9.45%, 6.83%, and 20.44%, respectively. In summary, these results confirm the successful formation of the CoNi_2_S_4_@g-C_3_N_4_ nanocomposite.

**Fig. 3:**
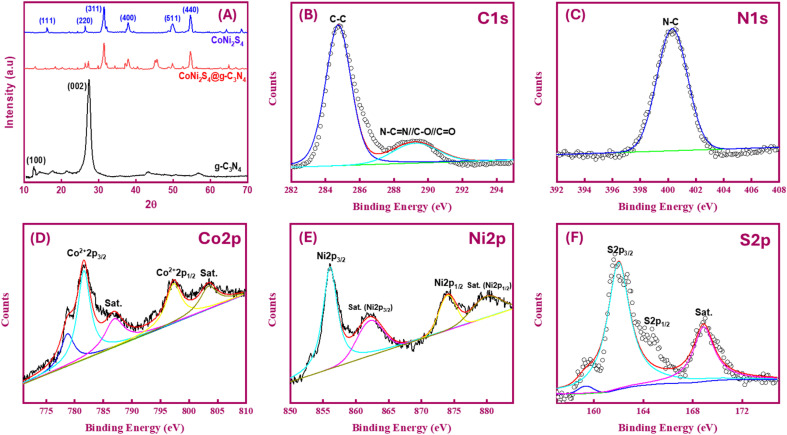
(A) XRD profiles of g-C_3_N_4_, CoNi_2_S_4_, and CoNi_2_S_4_@g-C_3_N_4_; XPS spectra of C 1s (B), N 1s (C), Co 2p (D), Ni 2p (E), and S 2p (F) of CoNi_2_S_4_@g-C_3_N_4_ sample.

The X-ray diffraction (XRD) patterns depicted in Fig. 3A were utilized for phase analysis of g-C_3_N_4_, CoNi_2_S_4_, and CoNi_2_S_4_@g-C_3_N_4_. The observed XRD peaks are consistent with the g-C_3_N_4_ phase (JCPDS 87–1526). Specifically, the notable peak at 27.45° typically assigned to the (002) plane of graphitic materials, while a minor diffraction peak at 12.54° corresponds to an interplanar (100) plane.^34^ Distinct peaks attributed to CoNi_2_S_4_ are observed at 16.2°, 26.36°, 31.5°, 37.9°, 49.8°, and 54.67°, which align well with the (111), (220), (311), (400), (511), and (440) facets of the cubic phase CoNi_2_S_4_ structure, as per standard pattern JCPDS no. 24-0334.^26,35^ Furthermore, the XRD pattern of the CoNi_2_S_4_@g-C_3_N_4_ composite exhibits integrated peaks from both g-C_3_N_4_ and CoNi_2_S_4_, confirming the successful creation of the CoNi_2_S_4_@g-C_3_N_4_ nanocomposite.

X-ray photoelectron spectroscopy (XPS) was utilized to investigate the chemical bonding, chemical composition, and valence states of elemental components. The XPS survey spectra indicated the presence of C, N, O, and trace amounts of S in the g-C_3_N_4_ sample (Fig. S2A†). The origin of the oxygen and sulfur is likely from the urea and thiourea used in the synthesis process. High-resolution XPS spectra for the C 1s and N 1s regions of g-C_3_N_4_ showed distinct peaks at binding energies (BE) of 284.77 eV and 288.25 eV, which correspond to sp^2^ C–C and N–C=N bonding, respectively (Fig. S2B†). The N 1s spectrum exhibited peaks at BEs of 398.79 eV and 401.1 eV, characteristic of C–N–H and C–N=N bonds, respectively (Fig. S2C†). These spectral features are consistent with those of pure g-C_3_N_4_, thereby confirming the successful synthesis of g-C_3_N_4_.^36,37^ For the CoNi_2_S_4_@g-C_3_N_4_ sample, the C 1s spectrum exhibits peaks at BE of 289.25 eV and 284.75 eV, while the N 1s spectrum displays peaks at BE of 400.3 eV (Fig. 3B and C). Some shifts in BE values are observed, likely due to the grafting of CoNi_2_S_4_ onto the g-C_3_N_4_ surface. In the Co 2p XPS spectrum (Fig. 3D), the peaks at 778.8 and 793.8 eV for Co 2p_3/2_ and Co 2p_1/2_ are characteristic peaks of Co^3+^. While the remaining peaks at 781.5 and 797.9 eV for Co 2p_3/2_ and Co 2p_1/2_ are attributed to the Co^2+^ spin–orbits. The corresponding satellite peaks can be observed at 803.5 eV (Co 2p_1/2_) and 787.1 eV (Co 2p_3/2_).^38^ In the Ni 2p XPS spectrum (Fig. 3E), strong peaks at 856.02 and 874.06 eV for Ni 2p_3/2_ and Ni 2p_1/2_ are detected, signifying the presence of Ni^3+^. The corresponding satellite heights can be observed at 862.24 eV (Ni 2p_3/2_) and 880.23 eV (Ni 2p_1/2_).^35^ In the S 2p XPS spectrum (Fig. 3F), the BEs at 162 and 164.6 eV are attributed to S 2p_3/2_ and S 2p_1/2_ core levels, respectively. The peak at 162.3 eV is typically assigned to the M^*n*+^–S bond in CoNi_2_S_4_ material. Additionally, the other peak at 164.6 eV is assigned to the sulfur ions in low coordination, which is commonly reported to S-vacancies in the material matrix. The highest detected at 168.87 eV is attributed to the shakeup satellite.^39,40^

N_2_ ads–des isotherm analysis was done to evaluate pore features and surface area of the g-C_3_N_4_ and CoNi_2_S_4_@g-C_3_N_4_ adsorbents. The resulting curves displayed distinct ads/des inflexions and hysteresis loops at *P*/*P*_0_ ranging from ∼0.4 to ∼0.9, indicating the porosity of the studied adsorbents (Fig. S3A†). The application of the Brunauer–Emmett–Teller (BET) model revealed a specific surface area of 63.18 m^2^ g^−1^ for g-C_3_N_4_, which decreased to 8.95 m^2^ g^−1^ for the CoNi_2_S_4_@g-C_3_N_4_ sample due to the incorporation of CoNi_2_S_4_ within and around the pores. This incorporation process also resulted in a reduction of pore volume from 0.21 cm^3^ g^−1^ (for g-C_3_N_4_) to 0.033 cm^3^ g^−1^ (for CoNi_2_S_4_@g-C_3_N_4_). Further analysis using the Barrett–Joyner–Halenda (BJH) method elucidated the distribution of pore sizes in the adsorbents, with narrow peaks observed at 3.02 nm and 2.89 nm, confirming the mesoporous nature of both g-C_3_N_4_ and CoNi_2_S_4_@g-C_3_N_4_ adsorbents (Fig. S3B†). These mesoporous characteristics are advantageous for efficiently trapping Au(iii) ions.

### 3.2. Au(iii) adsorption optimization

**Fig. 4:**
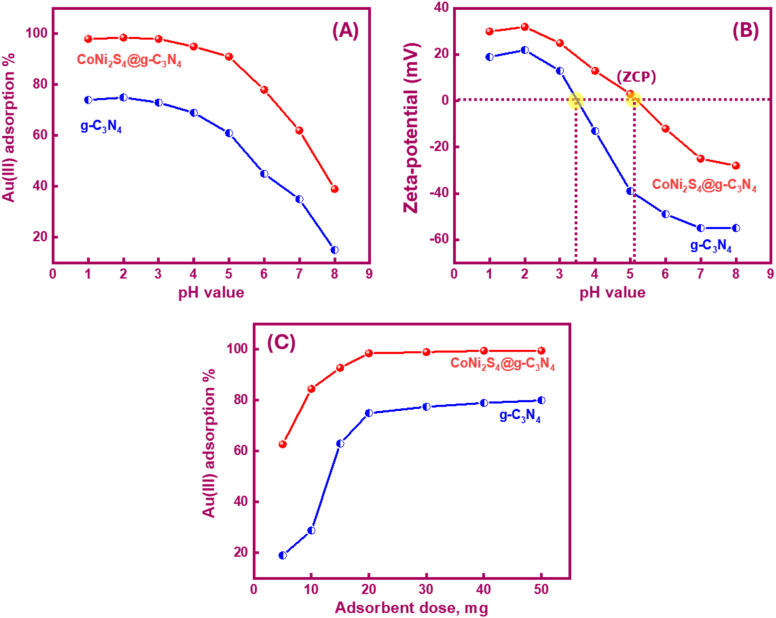
(A) Impact of pH value on the trapping performance of g-C_3_N_4_ and CoNi_2_S_4_@g-C_3_N_4_ adsorbents; (B) zeta-potential profile of g-C_3_N_4_ and CoNi_2_S_4_@g-C_3_N_4_ adsorbents based on the change of pH value; (C) effect of –C_3_N_4_ and CoNi_2_S_4_@g-C_3_N_4_ dose (mg) on their adsorption performance.

The pH significantly influenced the Au(iii) trapping. Moreover, the selectivity of the adsorbents for Au(iii) ions could be controlled by adjusting the pH value. Variations in pH affected the adsorbent active sites' charge, leading to either improved or demoted Au(iii)-trapping efficiency. In the bench-top trials, 20 mg of g-C_3_N_4_ and CoNi_2_S_4_@g-C_3_N_4_ adsorbents were stirred with 25 mL of 30 ppm Au(iii) solution for 60 minutes over a pH range of 1 to 8 at room temperature. Data in Fig. 4A revealed that 75% and 98.5% of Au(iii) could be trapped at pH 2.0 using g-C_3_N_4_ and CoNi_2_S_4_@g-C_3_N_4_, respectively. The trapping efficiencies of both adsorbents improved faintly as the pH rose from 1 to 2. In an acidic medium (pH 2), Au(iii) was present as a negative AuCl_4_^−^ species. The attraction forces between the active sites of g-C_3_N_4_ and CoNi_2_S_4_@g-C_3_N_4_ and AuCl_4_^−^ species were mainly induced by the pH environment. At pH 2, the highest positive charge of g-C_3_N_4_ and CoNi_2_S_4_@g-C_3_N_4_ adsorbents was observed (+22 mV to +32 mV), indicating surface protonation (Fig. 4B). The high protonation (H^+^) at pH 2 boosted the attraction between negatively AuCl_4_^−^ ions and outer positively charged positions, thereby improving adsorption efficiency. As the pH increased, the surfaces of g-C_3_N_4_ and CoNi_2_S_4_@g-C_3_N_4_ began to acquire a negative charge, growing the repulsion between the negatively AuCl_4_^−^ species and the negative-charged sites. Additionally, Au(iii) may exist as [AuCl_3_OH]^−^ and [AuCl_2_(OH)_2_]^−^ species, creating an electrostatic repulsion behaviour between the anionic Au(iii) species and the negative-charged centres.^41,42^

Fig. 4B indicated that the zero-charge points (ZCP) of g-C_3_N_4_ and CoNi_2_S_4_@g-C_3_N_4_ adsorbents were found to be at pH 3.5 and 5.1, respectively. At pH < pH_ZCP_, the active sites on the surfaces of g-C_3_N_4_ and CoNi_2_S_4_@g-C_3_N_4_ become positive-charged sites due to the creation of protonated functional groups. Conversely, at pH > pH_ZCP_, active sites acquire negative charges because of the creation of negatively charged groups. The zeta-potential data proved the highest Au(iii)-trapping efficiency obtained at pH 2. The strong binding interactions at this pH ensured maximum adsorption and selectivity for Au(iii) ions. Subsequent studies on the trapping of Au(iii) ions were conducted using g-C_3_N_4_ and CoNi_2_S_4_@g-C_3_N_4_ under optimal pH conditions (*i.e.*, pH 2).

Different doses of g-C_3_N_4_ and CoNi_2_S_4_@g-C_3_N_4_ adsorbents (ranging from 5 to 50 mg) were employed to assess the effect of adsorbent dose on the adsorbent performance toward Au(iii)-trapping. As presented in Fig. 4C, the adsorption efficiency of g-C_3_N_4_ and CoNi_2_S_4_@g-C_3_N_4_ adsorbents increased with the growth of adsorbents quantity. This increase suggests that the adsorption efficiencies of g-C_3_N_4_ and CoNi_2_S_4_@g-C_3_N_4_ are dependent on the availability of active surface sites, and larger quantities of adsorbents provide additional internal and external active sites along the adsorbent surfaces, thereby enhancing efficiency. When the adsorbent quantity was insufficient, the number of interior and exterior surface-active sites was less than what was required for whole trapping. Consequently, 20 mg of g-C_3_N_4_ and CoNi_2_S_4_@g-C_3_N_4_ adsorbents were found to be enough and suitable for following adsorption experiments.

### 3.3. Adsorption isotherm study

**Fig. 5:**
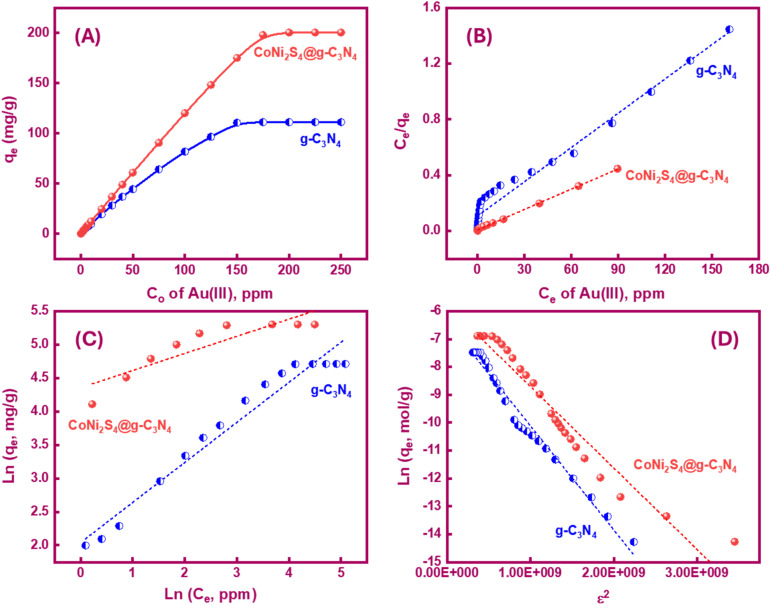
(A) Effect of preliminary [Au(iii)] on the *q*_e_ values of g-C_3_N_4_ and CoNi_2_S_4_@g-C_3_N_4_ adsorbents, and linear plots of Langmuir (B), Freundlich, and (C) Dubinin–Radushkevich (D–R) (D) isotherm models.

A series of bench-top tests were conducted to evaluate the impact of initial Au(iii) concentrations (ranging from 0.1 to 250 ppm) on the adsorption capacities of g-C_3_N_4_, CoNi_2_S_4_, and CoNi_2_S_4_@g-C_3_N_4_ adsorbents under optimal conditions. As shown in Fig. 5A, the adsorption capacities (*q*_e_, mg g^−1^) of g-C_3_N_4_ and CoNi_2_S_4_@g-C_3_N_4_ increased sharply at low Au(iii) concentrations before reaching equilibrium at higher concentrations. This rapid increase at low concentrations is indicative of type I sorption on porous adsorbents, according to the Langmuir classification. The maximum experimental adsorption capacities were found to be 111.25 mg g^−1^ for g-C_3_N_4_ and 200.6 mg g^−1^ for CoNi_2_S_4_@g-C_3_N_4_. Fig. S4† presents the Au(iii) adsorption capacity of the CoNi_2_S_4_ sample, which was determined to be 132.86 mg g^−1^. Although this is a significant capacity, it is lower than the 200.6 mg g^−1^ achieved by the CoNi_2_S_4_@g-C_3_N_4_ nanocomposite. This comparison underscores the enhancement in adsorption performance when CoNi_2_S_4_ is incorporated into the g-C_3_N_4_ matrix. The g-C_3_N_4_ support increases the surface area, improves the dispersion of CoNi_2_S_4_ particles, and provides additional active sites for adsorption, collectively boosting the overall adsorption capacity and efficiency. The synergistic interaction between CoNi_2_S_4_ and g-C_3_N_4_ in the nanocomposite results in superior adsorption kinetics and higher selectivity for Au(iii) ions, highlighting the crucial role of the g-C_3_N_4_ support in the composite material.

Langmuir and Freundlich isotherm models were employed to discover the nature of Au(iii)-adsorbents interaction. Additionally, these models were used to determine the theoretical adsorption capacity of g-C_3_N_4_ and CoNi_2_S_4_@g-C_3_N_4_ adsorbents, based on the following equations:^43,44^
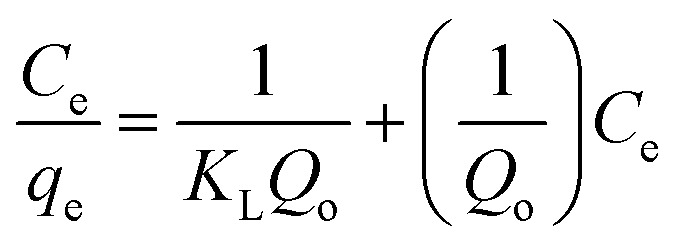

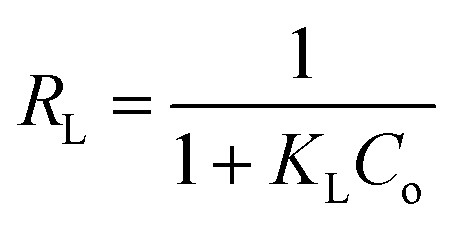

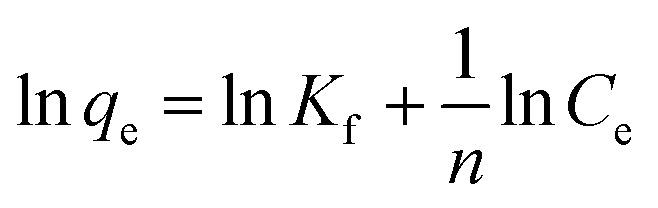


*Q*_o_, *K*_L_ and *K*_f_ are the theoretical adsorption capacities of g-C_3_N_4_ and CoNi_2_S_4_@g-C_3_N_4_ adsorbents (mg g^−1^), Langmuir constant (L mg^−1^), and Freundlich constant. And *n* represents the sorption intensity. The coefficient of determination (*R*^2^) for the linear relationships confirmed that the Langmuir model is more appropriate than the Freundlich model (Fig. 5B and C), indicating the creation of chemical bonds between Au(iii) ions and active sites of the g-C_3_N_4_ and CoNi_2_S_4_@g-C_3_N_4_ in a single layer. The *Q*_o_, *K*_L_, *K*_F_, and *n* values were determined and listed in Table 1. The theoretical *Q*_o_ values for g-C_3_N_4_ and CoNi_2_S_4_@g-C_3_N_4_ adsorbents were 121.95 mg g^−1^ and 205.34 mg g^−1^, respectively, which aligned well with the experimental results. Additionally, 1/*n* < 1 confirmed the chemical interaction between Au(iii) ions and used adsorbents. Findings specified that the g-C_3_N_4_ and CoNi_2_S_4_@g-C_3_N_4_ adsorbents are effective at both low and high Au(iii) concentration levels.

**Table 1:** Isotherm and kinetic parameters of Au(iii)-sorption using g-C_3_N_4_ and CoNi_2_S_4_@g-C_3_N_4_

	Langmuir model		Freundlich model		DR		PFO		PSO		IPD	
g-C_3_N_4_	Experiential *q*_m_ (mg g^−1^)	111.25			*R* ^2^	0.97						
	*R* ^2^	0.97	*R* ^2^	0.95	*q*_o_ mol g^−1^	0.0017	*R* ^2^	0.65	*R* ^2^	0.98	*R* ^2^	0.654
	*Q*_o_	121.95	*K*_F_, mg g^−1^	7.69	*δ* mol^2^ J^−2^	3.7 × 10^−9^	*q*_e_, mg g^−1^	100.69	*q*_e_, mg g^−1^	32.5	*I*	12.65
	*K*_L_, L mg^−1^	0.076	n	1.66	*E* J mol^−1^	11 624.76	*K*_1_, min^−1^	0.0033	*K*_2_, g mg^−1^ min^−1^	0.00313	*K*_id_ mg g^−1^ min^−0.5^	2.02
					*E* kJ mol^−1^	11.624						
CoNi_2_S_4_@g-C_3_N_4_	Experiential *q*_m_ (mg g^−1^)	200.6			*R* ^2^	0.93						
	*R* ^2^	0.998	*R* ^2^	0.77	*q*_o_ mol g^−1^	0.0031	*R* ^2^	0.45	*R* ^2^	0.999	*R* ^2^	0.64
	*Q*_o_	205.34	*K*_F_, mg g^−1^	77.47	*δ* mol^2^ J^−2^	2.92 × 10^−9^	*q*_e_, mg g^−1^	181.97	*q*_e_, mg g^−1^	38.46	*I*	30.6
	*K*_L_, L mg^−1^	0.611	*n*	3.85	*E* J mol^−1^	13 085.6	*K*_1_, min^−1^	0.0019	*K*_2_, g mg^−1^ min^−1^	0.0124	*K*_id_ mg g^−1^ min^−0.5^	0.843
					*E* kJ mol^−1^	13.085						

Furthermore, the Dubinin–Radushkevich (DR) isotherm model characterize of the adsorption process based on the porous structure of g-C_3_N_4_ and CoNi_2_S_4_@g-C_3_N_4_ adsorbents:^45,46^ln *q*_e_ = ln *q*_o_ − *δε*^2^
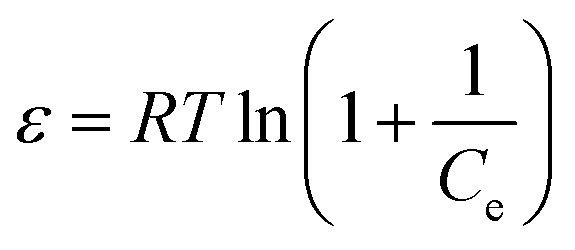
where *ε* is the Polanyi potential, *C*_e_ (mol L^−1^) is the equilibrium Au(iii)-concentration, *q*_o_ (mol g^−1^) represents the maximum Au(iii) adsorption capacity, and *δ* (mol^2^ J^−2^) is the constant related to the Au(iii)-sorption energy. The Au(iii)-sorption energy (*E*, J mol^−1^) offers insight into the Au(iii)-sorption nature. The value of *E* can be concluded *via* the subsequent relationship:^47^
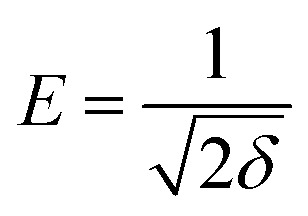


If the *E* value is <8 kJ mol^−1^, the sorbate–sorbent interaction is classified as physisorption, while *E* value ranging from 8 to 16 kJ mol^−1^ signifies a chemisorption process. In the current study, the *E* values for g-C_3_N_4_ and CoNi_2_S_4_@g-C_3_N_4_ sorbents were estimated as 11.624 and 13.085 kJ mol^−1^, confirming the chemical nature of the Au(iii) adsorption process (Fig. 5D and Table 1).

### 3.4. Kinetic study of Au(iii) adsorption

**Fig. 6:**
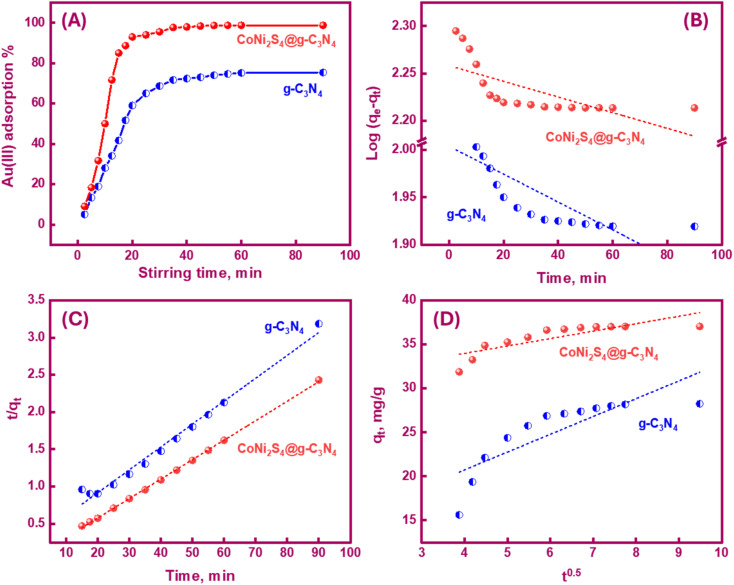
Effect of stirring time on the trapping efficiency of Au(iii) adsorption using g-C_3_N_4_ and CoNi_2_S_4_@g-C_3_N_4_ adsorbents, and linear plots of PFO (B), PSO (C), and intra-particle diffusion (D) kinetic models under the best trapping conditions.

Determining the required time for the Au(iii)-trapping process is economically important to achieve maximum adsorption efficiency within the lowest consumed time as much as possible. So, numerous stirring times (2.5–90 minutes) were tested to explore the impact of stirring time on Au(iii)-sorption efficiency. Outcomes in Fig. 6A specified that the Au(iii)-trapping efficiency encouraged with longer stirring times, reaching 75% and 98.5% for g-C_3_N_4_ and CoNi_2_S_4_@g-C_3_N_4_, respectively, within 60 minutes. Thus, equilibrium could be achieved within 60 minutes. Pseudo-first/second-order (PFO & PSO) kinetic models were employed to investigate the sorption interaction mechanism. The PFO and PSO kinetic equations are described as follows:^48^
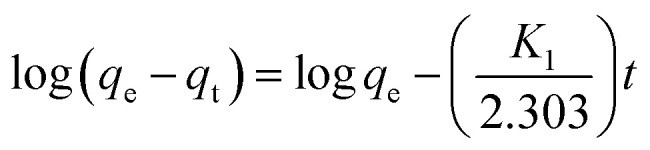

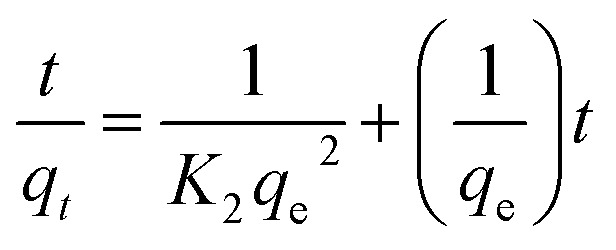


*K*_1_ (min^−1^) and *K*_2_ (g mg^−1^ min^−1^) are the rate constants of PFO and PSO models. *q*_e_ and *q*_*t*_ represent the quantity of trapped Au(iii) ions (mg g^−1^) at equilibrium and at time *t*. The constants can be determined from the slope and intercept of the linear plots of log(*q*_e_ − *q*_*t*_) and *t*/*q*_*t*_ against *t* (Fig. 6B and C). The results indicated that the PSO model was more appropriate for illustrating the kinetic pathway of Au(iii) adsorption onto g-C_3_N_4_ and CoNi_2_S_4_@g-C_3_N_4_, suggesting a chemisorption mechanism. The *K*_2_ value for the CoNi_2_S_4_@g-C_3_N_4_ adsorbent (0.0124) was greater than that of the g-C_3_N_4_ adsorbent (0.00313) (Table 1), indicating that CoNi_2_S_4_@g-C_3_N_4_ has a significantly higher adsorption rate for Au(iii) ions. This implies that the composite material CoNi_2_S_4_@g-C_3_N_4_ enhances the adsorption kinetics, likely due to improved surface properties, increased active sites, or better interaction between sorbents and Au(iii) ions.

The Au(iii)-sorption process typically progresses through multiple stages, including adsorbate transfer, external prevalence, intra-particle diffusion (IPD), and eventually chemical or physical interaction between the sorbents' active sites and Au(iii) ions. The IPD model assumes that the prevalence of Au(iii) is the rate-controlling step during the adsorption procedure, and the diffusion direction can vary. This kinetic model was used to evaluate the IPD rate constant. The IPD equation can be represented as next:^49^
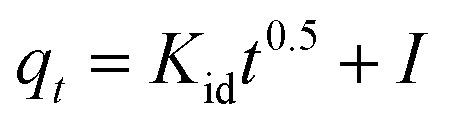


*K*_id_ (mg g^−1^ min^−0.5^) is the rate constant of the IPD. The intercept *I* of the IPD equation depends on the thickness of the external boundary layer. The plot of *q*_*t*_*versus t*^0.5^ for g-C_3_N_4_ and CoNi_2_S_4_@g-C_3_N_4_ adsorbents is shown in Fig. 6D. Table 1 shows the calculated values of *R*^2^, *K*_id_, and *I*. For CoNi_2_S_4_@g-C_3_N_4,_ the *K*_id_ value is lower than that for g-C_3_N_4_, suggesting that the diffusion of Au(iii) along CoNi_2_S_4_@g-C_3_N_4_ matrix is slower than g-C_3_N_4_. This may be due to the dense grafting of CoNi_2_S_4_ along the g-C_3_N_4_ surface and pores, facilitating quick access to active sites. A higher *I* value enhances the influence of the outer surface prevalence, improving the rate of the Au(iii)-sorption process. Rapid sorption of Au(iii) ions using g-C_3_N_4_ and CoNi_2_S_4_@g-C_3_N_4_ adsorbents occurred within the first 60 minutes, primarily due to the prevalence of the external layer or mesopores.

### 3.5. Selectivity study of used adsorbents

**Fig. 7:**
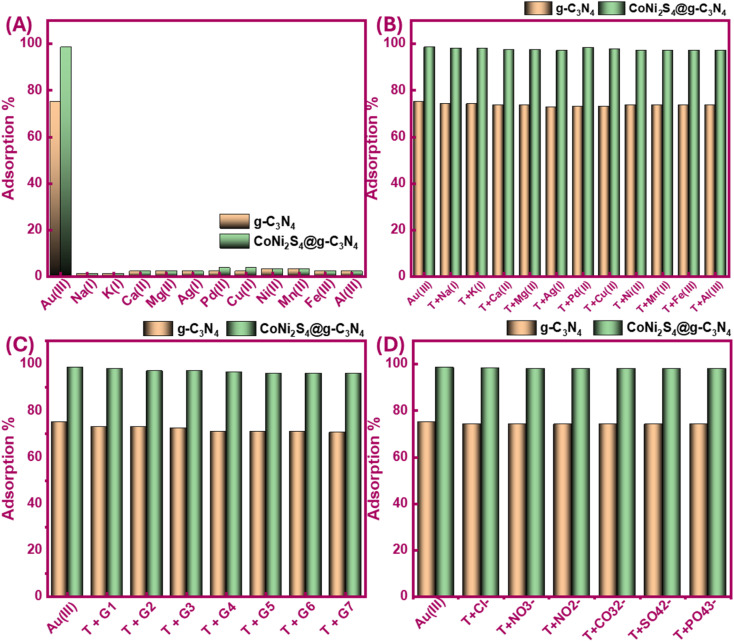
Selectivity profile of g-C_3_N_4_ and CoNi_2_S_4_@g-C_3_N_4_ to adsorb Au(iii) ions and other co-existing ions in single (A), binary (B), and group (more than one competitive ion) systems (C), and anions (D) systems at optimum trapping conditions. G1{Au(iii) + Ag(i)}, G2{ Au(iii) + Cu(ii)}, G3{ Au(iii) + Mn(ii) + Al(iii)}, G4{ Au(iii) + Co(ii) + Hg(ii) + Pb(ii)}, G5{ Au(iii) + Ni(ii) Ca(ii) + Mn(ii) + Fe(iii)}, G6{ Au(iii) + Al(iii) + Mg(ii) + Pb(ii) + Hg(ii) + Cr(iii)}, G7{ Au(iii) + Hg(ii) + Cd(ii) + K(i) + Pd(ii) + Fe(iii)}.

Selectivity is a critical factor in metal extraction processes, as it aims to isolate specific ions in their pure states from complex mixtures containing various ions. Here, we investigated the impact of competing ions on the Au(iii)-trapping efficiency in both single and combined systems. At the optimal pH of 2, 20 mg of g-C_3_N_4_ and CoNi_2_S_4_@g-C_3_N_4_ were stirred with 25 mL of a 30 ppm Au(iii) solution, along with 30 ppm of Na(i), K(i), Ag(i), Mg(ii), Ca(ii), Pd(ii), Mn(ii), Fe(iii), Cu(ii), Ni(ii), and Al(iii). As shown in Fig. 7A, the adsorbents demonstrated a high selectivity for Au(iii), with minimal adsorption of other cations. The solution's pH was a significant determinant of this selectivity. In acidic conditions, the high concentration of H^+^ ions hindered the adsorption of other cations. Furthermore, when Au(iii) was presented alongside another metal ion in binary systems and exposed to the adsorbents at pH 2, findings indicated that competing cations had a negligible impact on Au(iii) ions sorption (Fig. 7B).

Further batch experiments were conducted in a mixture system using more than one cation with Au(iii) (G1 to G7), as explained in Fig. 7C caption. The findings in Fig. 7C demonstrated no noteworthy variation in Au(iii) adsorption, maintaining a high adsorption efficiency of 96–98%, despite the presence of increased concentrations of diverse cations. Overall, both g-C_3_N_4_ and CoNi_2_S_4_@g-C_3_N_4_ exhibited strong anti-cation interference capabilities, effectively capturing Au(iii) with high selectivity. We also evaluated the impact of co-existing anions on the adsorption behavior of Au(iii). Fig. 7D shows that the Au(iii)-trapping efficiency of g-C_3_N_4_ and CoNi_2_S_4_@g-C_3_N_4_ decreased slightly by 1–2% in the company of Cl^−^, NO_3_^−^, NO_2_^−^, SO_4_^2−^, CO_3_^2−^, and PO_4_^3−^ ions. This minor reduction in adsorption efficiency suggests a relatively small adverse impact of these anions on Au(iii)-sorption performance. Despite this minor interference, the robust binding between negatively Au(iii) species and positive-charged sites of g-C_3_N_4_ and CoNi_2_S_4_@g-C_3_N_4_ at pH 2 remained dominant.

### 3.6. Recycling and reusability of spent extractors

**Fig. 8:**
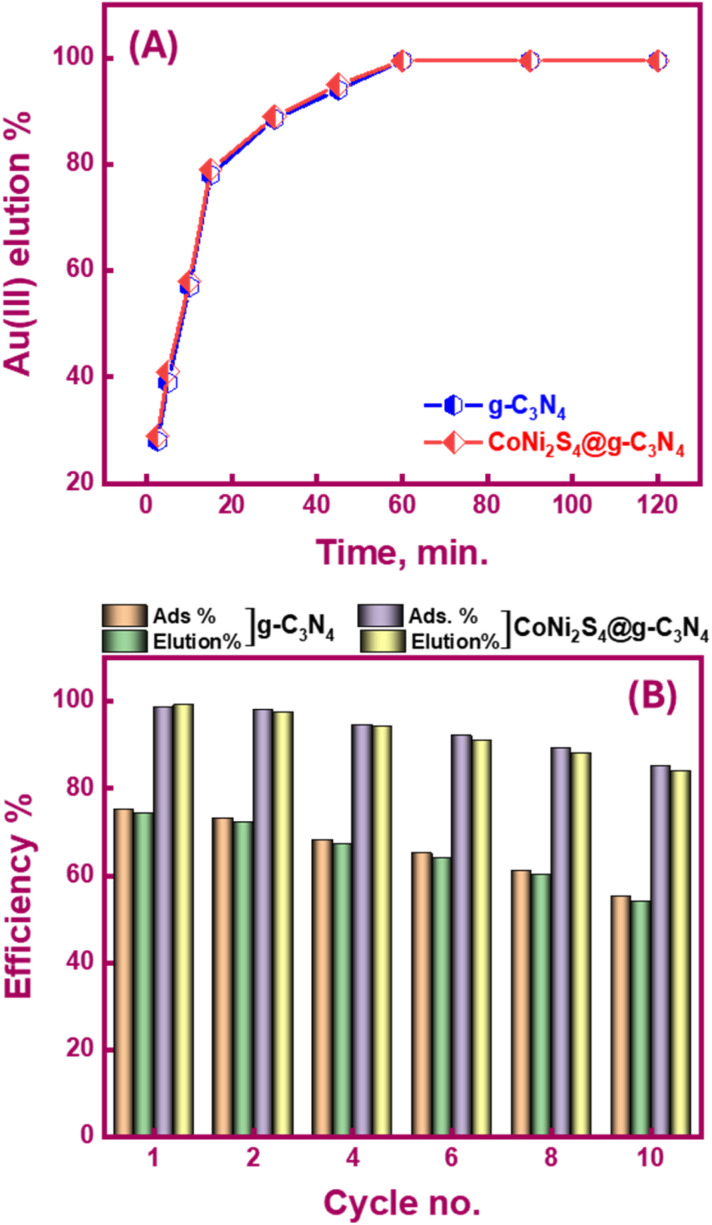
(A) Influence of stirring time on the elution efficacy of Au(iii) ions from used g-C_3_N_4_ and CoNi_2_S_4_@g-C_3_N_4_ adsorbents; (B) reusability of g-C_3_N_4_ and CoNi_2_S_4_@g-C_3_N_4_ adsorbents under the optimum adsorption and elution circumstances.

Spent g-C_3_N_4_ and CoNi_2_S_4_@g-C_3_N_4_ adsorbents can be recycled using a batch elution/desorption protocol, minimizing the cost of the extraction process and the generated further waste. Elution is essential for obtaining target ions in their pure forms. In bench-top trials, Au(iii)-loaded adsorbents were treated with NaOH and thiourea (0.1 M) under continuous stirring to investigate the effect of elution time (Fig. 8A). Thiourea forms a stable complex with Au(iii) ions {[Au(CS(NH_2_)_2_)_2_]^+^}in the presence of NaOH, facilitating the desorption of gold from the adsorbent. NaOH adjusts the pH and provides an alkaline environment that enhances the desorption efficiency of the thiourea complex. Results showed that over 99% of the loaded Au(iii) were recovered within 60 minutes. The treated g-C_3_N_4_ and CoNi_2_S_4_@g-C_3_N_4_ adsorbents were then filtered, dried, and reused for subsequent Au(iii) adsorption. Fig. 8B explains that the recycled g-C_3_N_4_ and CoNi_2_S_4_@g-C_3_N_4_ adsorbents could be reused for ten cycles. After the tenth cycle, the adsorption efficiency of g-C_3_N_4_ and CoNi_2_S_4_@g-C_3_N_4_ adsorbents decreased to 55% and 85%, respectively, while the elution efficacy stayed high (>99%). Despite the decrease in adsorption efficiency due to the stripping agent's adverse effects during repeated operations, which may destroy some of the surface-active sites. The findings indicate that the CoNi_2_S_4_@g-C_3_N_4_ adsorbent is effective in e-waste treatment and Au(iii) ion recovery. This is attributed to its superior efficacy, cost-efficiency, reusability, and robustness even after multiple reuse cycles.

### 3.7. Au(iii) extraction from SMB

Recycling e-waste has garnered global attention as a sustainable strategy for accessing precious metals like gold while mitigating environmental impact. Our study focuses on e-waste recycling using highly selective extractors through a straightforward, rapid, and efficient approach. In this investigation, we utilized mesoporous CoNi_2_S_4_@g-C_3_N_4_ adsorbent to selectively capture Au(iii) ions amidst competitive ions from actual leachate. The extraction of Au(iii) from SMB comprised multiple phases: (i) leaching step to release Au(iii) ions from SMB to solutions, (ii) adsorption step to adsorb Au(iii) ions using CoNi_2_S_4_@g-C_3_N_4_ adsorbent under the optimum adsorption conditions, and (iii) elution step to collect Au(iii) ions in a pure form.

**Fig. 9:**
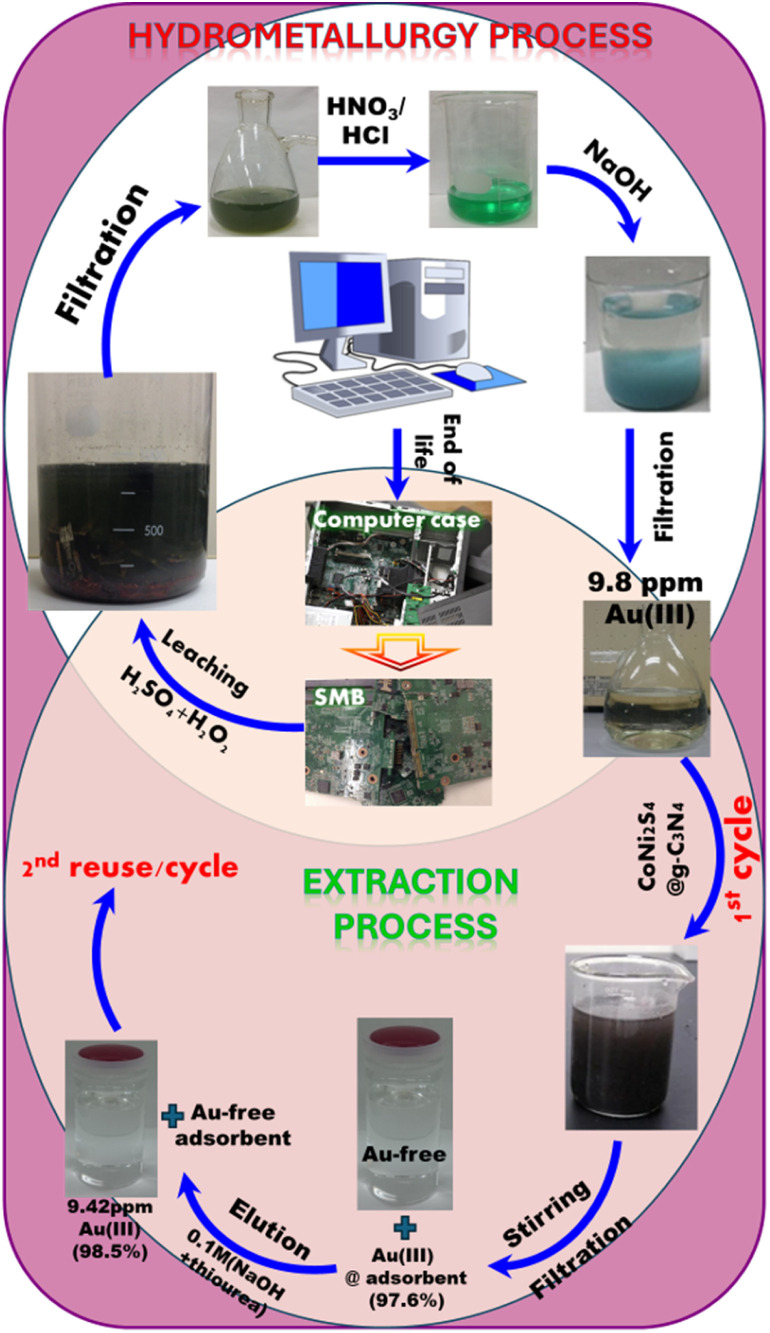
SMB hydrometallurgical processing and extraction process scheme of the Au(iii) ions from the SMBs using mesoporous CoNi_2_S_4_@g-C_3_N_4_ adsorbent.

The leaching process for Au(iii) ion releasing from SMB can be performed using a hydrometallurgical approach, which could be done through the next steps (see Fig. 9):

(i) Gathering SMBs: SMBs were isolated from obsolete computers and crushed into tiny fragments to ease the leaching process.

(ii) Leaching SMBs: Here, 100 g of crushed SMB was stirred overnight with a mixture of H_2_SO_4_ (5 M) and H_2_O_2_ (30%) to leach metals such as Ni(ii), Fe(iii), Al(iii), Zn(ii), and Cu(ii) ions. The remaining solid components were then filtered and separated.

(iii) Dissolving gold ions: The resulting solution was stirred with HNO_3_/HCl solution (1 : 3 v : v), leading to a soluble solution of Au(iii) ions and other ions.

(iv) Precipitation of other ions: Fe(iii), Sn(iv), and Al(iii) ions were precipitated using NaOH by adjusting the pH to 4–4.5, while Ag(i) ions were separated using NaCl as AgCl. Precipitates were then eliminated through centrifuge filtration.

(v) The obtained clear solution was employed for selective trapping of Au(iii) ions using the CoNi_2_S_4_@g-C_3_N_4_ adsorbent.

As shown in Table 2, Au(iii) ions and other competitive ions concentrations in the resulting liquor were quantified using Atomic Absorption Spectroscopy before interaction with the CoNi_2_S_4_@g-C_3_N_4_ adsorbent. The CoNi_2_S_4_@g-C_3_N_4_ adsorbent achieved an adsorption efficiency of 97.6%, and the elution efficiency for these adsorbents was 98.5%. This study demonstrates that the developed CoNi_2_S_4_@g-C_3_N_4_ adsorbent is highly effective in extracting, recovering, and purifying Au(iii) from obsolete SMB. These findings suggest the potential application of CoNi_2_S_4_@g-C_3_N_4_ adsorbents in environmental cleanup and e-waste management, offering a sustainable solution for recovering valuable Au(iii) ions from e-waste sources selectively.

**Table 2:** Selective adsorption study of Au(iii) ions using CoNi_2_S_4_@g-C_3_N_4_ adsorbent at specific extraction/elution conditions

[Coexisted metal ions] (ppm)	[Au(iii)] (ppm)	[Au(iii)], after adsorption (ppm)	[Au(iii)], after elution (ppm)	Adsorption %	Elution %
Ag(i) 0.06, Pd(ii) 0.12, Cu(ii) 5.25, Ni(ii) 1.7, Mn(ii) 0.07, Fe(iii) 9.3, Al(iii) 10.8, Zn(ii) 3.85, Pb(ii) 0.03, Hg(ii) 0.02, Cd(ii) 0.02	9.8	0.235	9.42	97.6	98.5

### 3.8. Advantages and potential limitations of the proposed method

In this section, we comprehensively compare our proposed method for Au(iii) extraction using the CoNi_2_S_4_@g-C_3_N_4_ nanocomposite with other established techniques, highlighting the specific advantages and potential limitations.

(I) Leaching process: pyrometallurgy involves high-temperature processing, which can efficiently extract gold and other metals from e-waste. However, these methods often require significant energy input, generate toxic by-products, and have lower selectivity, contaminating the recovered metals. In contrast, hydrometallurgy uses aqueous solutions to leach metals, which can be more environmentally friendly than pyrometallurgical methods. Therefore, we followed the hydrometallurgy method in the leaching process.

(II) Adsorption process: the CoNi_2_S_4_@g-C_3_N_4_ nanocomposite demonstrates a high adsorption capacity of 200.6 mg g^−1^, rapid adsorption kinetics, and excellent reusability, maintaining over 85% efficiency after ten cycles. These features make it a superior adsorbent compared to many conventional materials, as shown in Table 3. Table 3 compares the adsorption capacities of various adsorbents for extracting Au(iii) ions from aqueous solutions. The results demonstrate that CoNi_2_S_4_@g-C_3_N_4_ excels in Au(iii) adsorption, surpassing the performance of several other materials. These findings suggest that the CoNi_2_S_4_@g-C_3_N_4_ nanocomposite holds considerable promise for selectively capturing Au(iii) ions from e-waste.

**Table 3:** Comparing adsorption capacity for different materials that adsorb Au(iii) ions

Adsorbents	Adsorption capacity mg g^−1^	Ref.
g-C_3_N_4_	111.25	Here
CoNi_2_S_4_@g-C_3_N_4_	200.6	
Zr-MOF functionalized with mercapto-1,3,4-thiodiazole	301.5	50
UiO-66-NH_2_ modified by amidinothiourea	227.68	51
Dowex Marathon MSA commercial resin	73.53	52
Cellulose-based bio-adsorbent	5.07 mmol g^−1^	53
Humic acid	182.82	54
*N*-(2-[bis(2-aminoethyl)amino)ethyl]aminomethyl-polystyrene polymer bead	173.18	55
Imprinted ionic material SiO_2_(BGS/RHA)-TMPDT-Im-Au)	10.44	56
Ureido polymers containing large repeating ring	37.6	57
Clay mineral composite	108.3	58
Functionalized silica coating mercapto on iron sand magnetic material	125	59

(III) Specific advantages of our proposed method: (i) the CoNi_2_S_4_@g-C_3_N_4_ nanocomposite shows exceptional selectivity for Au(iii) ions, even in the presence of competing metal ions, and a high adsorption capacity, outperforming many traditional adsorbents; (ii) the process operates under mild conditions, reducing energy requirements and minimizing the use of hazardous chemicals. The reusability of the adsorbent further reduces operational costs and environmental impact; (iii) our method has been tested with actual e-waste leachates, demonstrating its effectiveness in real-world scenarios. The results indicate that the method can be easily scaled up for industrial applications, providing a viable solution for sustainable gold recovery.

(IV) Potential limitations: (i) using Co and Ni in the nanocomposite raises concerns about the sustainability and availability of these critical minerals. However, the high reusability and potential for recycling these materials mitigate this concern to some extent; (ii) while the adsorbent can be reused, the regeneration process must be optimized to ensure minimal loss of adsorption capacity and material integrity over multiple cycles.

## 4. Conclusion

In this study, we successfully synthesized a hybrid mesoporous nanocomposite of CoNi_2_S_4_@g-C_3_N_4_ for the selective recovery of Au(iii) ions from SMB. The CoNi_2_S_4_@g-C_3_N_4_ nanocomposite achieved a remarkable adsorption capacity of 200.6 mg g^−1^ at pH 2, significantly better than that of g-C_3_N_4_ alone (111.25 mg g^−1^). The high selectivity for Au(iii) ions over other competitive ions in both single and multi-ion systems underscores the potential of CoNi_2_S_4_@g-C_3_N_4_ as an effective adsorbent for gold recovery from e-waste. The adsorption process reached equilibrium within 60 minutes, fitting well with the PSO kinetic model. This rapid adsorption rate is advantageous for practical applications, where time efficiency is critical. The CoNi_2_S_4_@g-C_3_N_4_ nanocomposite exhibited excellent reusability, maintaining high adsorption efficiency (>85%) after ten cycles of adsorption and desorption. This recyclability, combined with the ease of synthesis, makes CoNi_2_S_4_@g-C_3_N_4_ a cost-effective and sustainable solution for industrial-scale gold recovery. In practical applications, the CoNi_2_S_4_@g-C_3_N_4_ nanocomposite demonstrated high efficiency in extracting Au(iii) ions from the leach liquor of SMB. The adsorption efficiency was 97.6%, with an elution efficiency of 98.5%, highlighting the nanocomposite's practical utility in e-waste management and resource recovery. In conclusion, the CoNi_2_S_4_@g-C_3_N_4_ nanocomposite presents a promising and viable adsorbent for the choosy recovery of Au(iii) from e-waste. Its high adsorption capacity, rapid kinetics, excellent selectivity, and reusability make it an attractive candidate for sustainable gold recovery processes. Future studies could focus on scaling up the synthesis process and exploring the application of CoNi_2_S_4_@g-C_3_N_4_ for recovering other valuable metals from various types of e-waste.

## Data availability

Relevant data are included in the paper and its ESI files.† Other data and parameters generated or analyzed during the study are available from the corresponding author upon reasonable request.

## Conflicts of interest

There are no conflicts to declare.

## Supplementary Material

RA-014-D4RA04476B-s001
