# Supplementary material for: pH-Dependent selective extraction of gold(iii) from synthetic solution and computer motherboard leachate using a hybrid nanocomposite
Source: RSC Adv. 2024 Jul 17;14(31):22569–81. doi: 10.1039/d4ra04476b (PMC11252653; doi:10.1039/d4ra04476b)
Supplement: RA-014-D4RA04476B-s001 [file RA-014-D4RA04476B-s001.pdf]

Electronic Supplementary Material (ESI) for RSC Advances.
This journal is © The Royal Society of Chemistry 2024

pH-dependent selective extraction of gold (III) from synthetic solution and computer motherboard leachate using a hybrid nanocomposite

Rabeea D. Abdel-Rahim, Mahmoud Thabet, Ahmed R. Abdellah, Mohamed O. Saleh, Ahmed M. M. Fadl, Adham M. Nagiub, Hassanien Gomaa*

Department of Chemistry, Faculty of Science, Al-Azhar University, Assiut 71524, Egypt.
* Corresponding author: H. Gomaa (h.gomaa@azhar.edu.eg)

Chemicals

The consumed substances were high quality, so we used them as obtained without any additional purification. Hydrontetrachlorogold ($HAuCl_4$), Cobalt(II) acetate ($Co(C_2H_3O_2)_2.4H_2O$), and nickel(II) acetate tetrahydrate ($Ni(C_2H_3O_2)_2.4H_2O$) were obtained from Innochem (Beijing) Technology Co., Ltd. Urea, thiourea, and polyvinylpyrrolidone (PVP, M wt. 55K) were attained from Shanghai Aladdin Bio-Chem Technology Co., Ltd.

Characterization Tools

The structural and compositional attributes of g-C_3N_4 and $CoNi_2S_4$@g-C_3N_4 were systematically characterized through a suite of analytical techniques. Scanning electron microscopy (SEM) investigations, conducted with a GAIA3 Tescan instrument at an electron acceleration voltage of 20 kV, were complemented by energy-dispersive X-ray spectroscopy (EDS) to elucidate the surface morphology and elemental composition. High-resolution transmission electron microscopy (HRTEM) was performed on a JEM-2100F microscope at 200 kV to provide detailed insights into the nanostructures of the materials. X-ray photoelectron spectroscopy (XPS) analyses were carried out using an ESCALAB 250XI instrument with an aluminum K-alpha X-ray source, set at 12 mA and 14.5 kV, to probe the surface chemical states and elemental composition. The crystalline phases of the samples were determined by X-ray diffraction (XRD) using a Rigaku SmartLab diffractometer, employing copper K-alpha radiation at a wavelength of 0.15406 nm, with the system operating at 40 kV and 150 mA. The porosity, including pore size distribution and specific surface area, was quantified through nitrogen adsorption-desorption isotherm measurements using a NOVA 2200e analyzer. Lastly, the surface charge characteristics of the materials were assessed using a Zetasizer Nano ZS (Model: ZEN 3600, Malvern, UK) to measure the zeta potential.

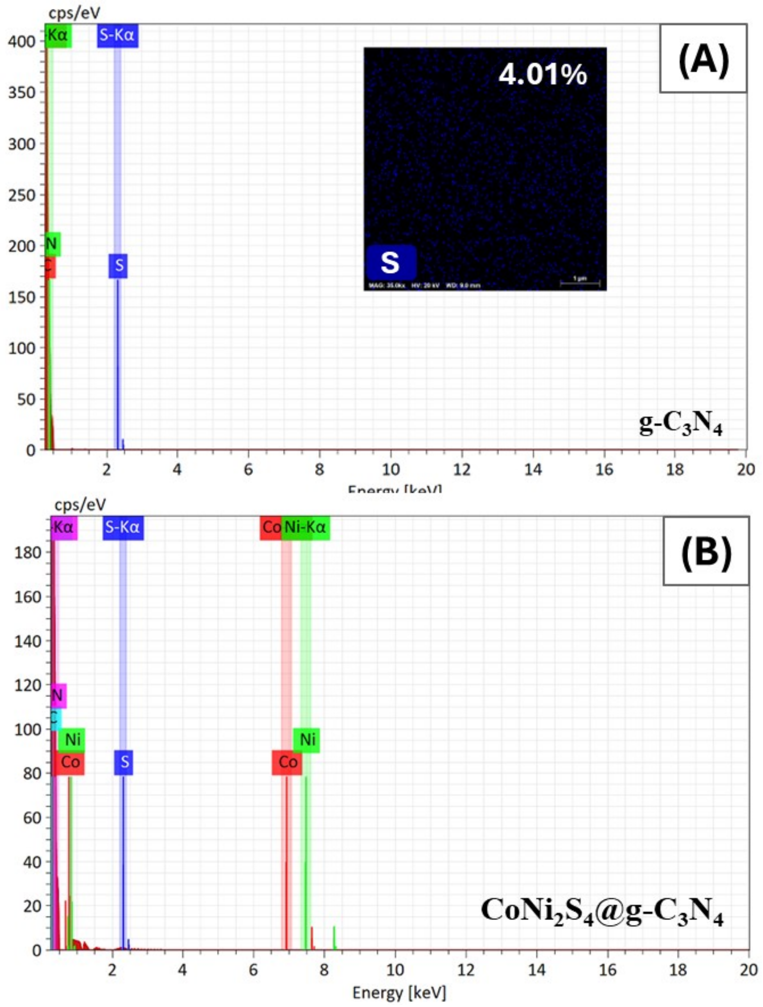

Figure S1. EDX patterns of g-C_3N_4 (A) and $CoNi_2S_4$@g-C_3N_4 (B) adsorbents.

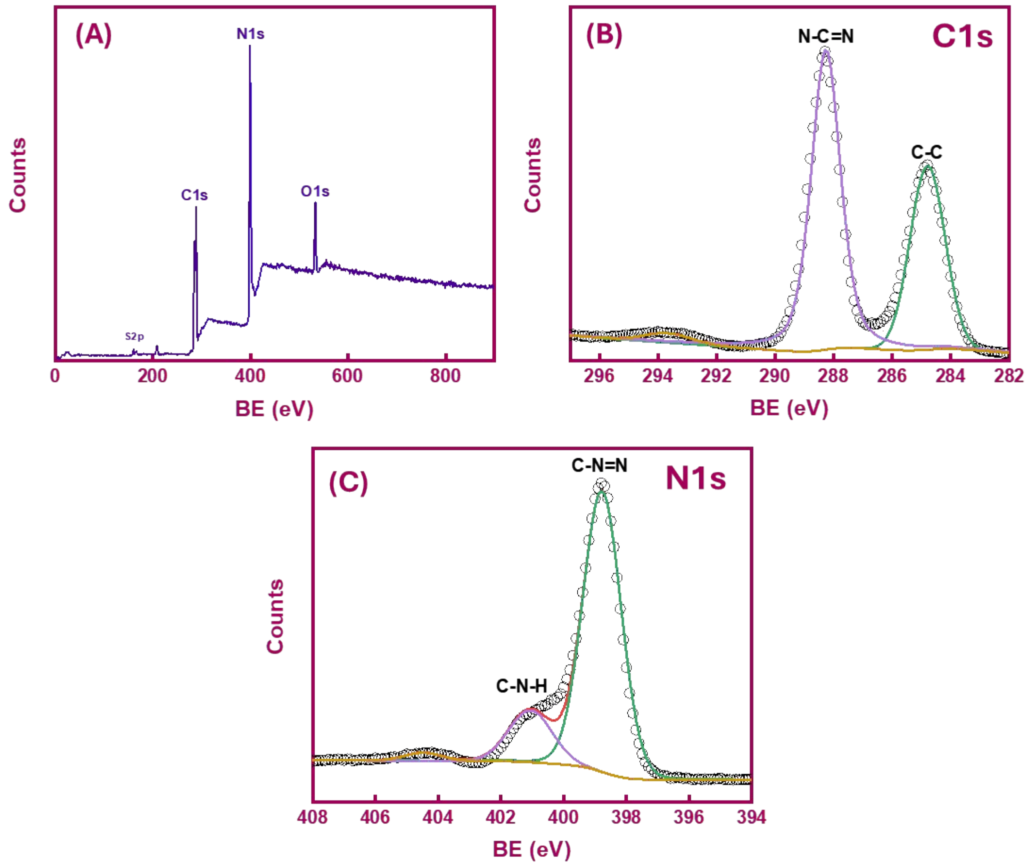

Figure S2. XPS survey (A) and spectra of C1s (B), and N1s (C) of g-C_3N_4 sample.

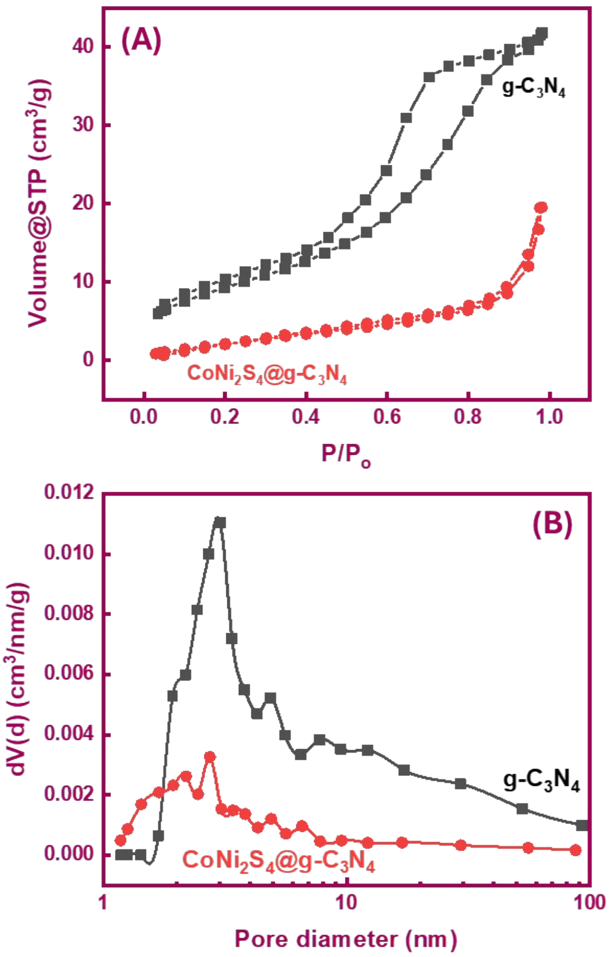

Figure S3. N_2-adsorption-desorption analysis (A) and BJH (B) of mesoporous g-C_3N_4 and $CoNi_2S_4$@g-C_3N_4 adsorbents.

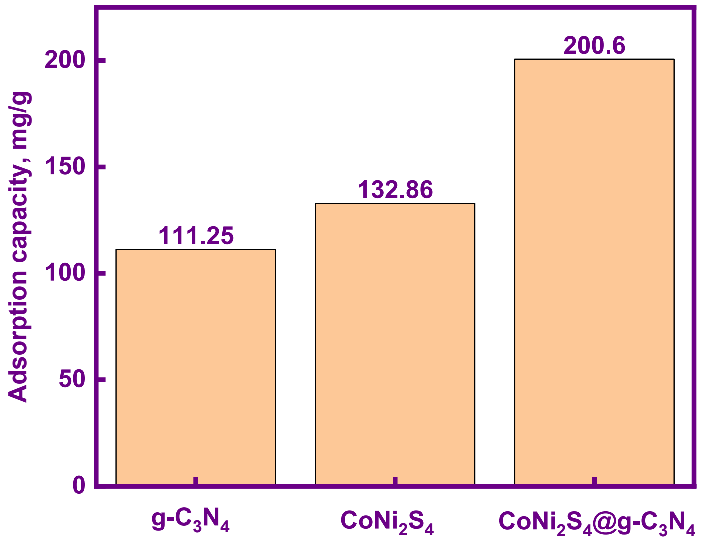

Figure S4. Au(III)-adsorption capacity of g-C_3N_4 $CoNi_2S_4$, and $CoNi_2S_4$@g-C_3N_4 nanocomposite at optimal adsorption conditions.
